# Sir Thomas Watson on the Treatment of Habitual Drunkards

**Published:** 1875-09

**Authors:** 


### Sir Thomas Watson on the Treatment of Habitual Drunkards.

Sir—My humble advocacy of this petition may be expressed in a very few sentences. I must preface them by saying that, although for much the greater part of my long professional life I was profoundly incredulous of the permanent reformation of habitual drunkards, facts have more recently come to my knowledge which have made me an almost sanguine convert to a better hope and belief.

Among habitual drunkards there are many in whom what was begun as a vice passes into a frightful bodily and mental disease. The frequent use of intoxicating liquors in excess, and especially of alcoholic drinks, leads at length to an accumulation of the specific poison of alcohol within the system, so that the bodily tissues, and chiefly, I suppose, the nervous tissues, which include the brain, become so impregnated, so charged with the poison, or so affected somehow as to produce a degree of craving which the unhappy dipso-maniac (for so he is rightly called) is utterly unable to resist or control. So imperative is this morbid craving that in some instances, by his own confession, he could not refrain from swallowing the customary stimulus, even if he were certain that death would be the result.

Now, of such persons it is found that, if they can be strictly debarred from all access to alcoholic drinks, they will surely, though slowly, recover from this form of mania; that the incorporated poison will be gradually dislodged and eliminated from the system by the silent and sole efficacy of that beneficent force which we medical men acknowledge so thankfully, to vis medicatrix naturæ; and the wretched man or woman will become once more able, and in no small percentage of cases willing, and even anxious, to abandon the vice, which had been the first step towards the maniacal disease.

Now, if this be so—and from all that I have seen, and heard, and read on the subject, from experience gathered on a large scale in America, from the teachings of some of our own lunatic asylums, and from the testimony of private observers, I am fully persuaded that it is so, I might even appeal on this point to some members of the deputation here present—then I conceive that the sanctioning, by some legislative measure, of retreats or reformatories, wherein, at the instance of his relations or friends, or by his own wish, or by the sentence of a magistrate, such a sufferer could be legally detained for a time (which has been estimated to be between three and twelve months, though in my judgment three months would be far too little, and of course ample provision should be made against any possible abuse of such detention), such legislative action, I say, could scarcely be regarded as anything less than a national blessing.—*British Medical Journal.*

